# Predicting potential drug-drug interactions by integrating chemical, biological, phenotypic and network data

**DOI:** 10.1186/s12859-016-1415-9

**Published:** 2017-01-05

**Authors:** Wen Zhang, Yanlin Chen, Feng Liu, Fei Luo, Gang Tian, Xiaohong Li

**Affiliations:** 1 State Key Lab of Software Engineering, Wuhan University, Wuhan, 430072 China; 2 School of Computer, Wuhan University, Wuhan, 430072 China; 3 School of Mathematics and Statistics, Wuhan University, Wuhan, 430072 China; 4 International School of software, Wuhan University, Wuhan, 430072 China

**Keywords:** Drug-drug interaction, Ensemble learning, Missing link prediction, Random walk

## Abstract

**Background:**

Drug-drug interactions (DDIs) are one of the major concerns in drug discovery. Accurate prediction of potential DDIs can help to reduce unexpected interactions in the entire lifecycle of drugs, and are important for the drug safety surveillance.

**Results:**

Since many DDIs are not detected or observed in clinical trials, this work is aimed to predict unobserved or undetected DDIs. In this paper, we collect a variety of drug data that may influence drug-drug interactions, i.e., drug substructure data, drug target data, drug enzyme data, drug transporter data, drug pathway data, drug indication data, drug side effect data, drug off side effect data and known drug-drug interactions. We adopt three representative methods: the neighbor recommender method, the random walk method and the matrix perturbation method to build prediction models based on different data. Thus, we evaluate the usefulness of different information sources for the DDI prediction. Further, we present flexible frames of integrating different models with suitable ensemble rules, including weighted average ensemble rule and classifier ensemble rule, and develop ensemble models to achieve better performances.

**Conclusions:**

The experiments demonstrate that different data sources provide diverse information, and the DDI network based on known DDIs is one of most important information for DDI prediction. The ensemble methods can produce better performances than individual methods, and outperform existing state-of-the-art methods. The datasets and source codes are available at https://github.com/zw9977129/drug-drug-interaction/.

**Electronic supplementary material:**

The online version of this article (doi:10.1186/s12859-016-1415-9) contains supplementary material, which is available to authorized users.

## Background

Drugs may interact when multiple drugs are co-prescribed. Drug-drug interactions (DDIs) may exert different effects, and adverse drug-drug interactions can lead to patient death or drug withdrawal [1–4]. DDI prediction can help to reduce unexpected effects as well as optimize the treatments in the drug design, clinical trials, and post-marketing surveillance.

Silico methods, in vitro methods, vivo experiments and clinical trials can identify DDIs, but they are labor-intensive and time-consuming. Statistical methods [5–9] were developed to detect whether the combination of two drugs is associated with an increased risk of certain adverse events, by analyzing spontaneous reports, insurance claim databases and electronic medical records.

In recent years, researchers collected drug data from literatures, reports and etc., and constructed public databases [10–17] which facilitate the development of computational prediction methods. To the best of our knowledge, a great number of machine learning methods were proposed to predict DDIs. Existing methods are roughly classified into two types: similarity-based methods and classification-based methods. The similarity-based methods employed the assumption that similar drugs may interact with a same drug. Gottlieb et al. [18] built prediction models by considering seven kinds of drug-drug similarities. Vilar et al. proposed the substructure similarity-based prediction method [19] and the interaction profile fingerprint similarity-based prediction method [20]. Li et al. [21] developed a Bayesian network of combining drug molecular similarity and phenotypic similarity to predict the combination efficacy of drugs. By using drug-drug similarity indirectly, Park et al. [22] applied a random walk with restart to simulate signaling propagation from drug targets and make predictions; Zhang et al. [23] adopted the label propagation method to build prediction models based on drug chemical substructures, drug side effects and drug and off side effects. Classification-based methods formulate the drug-drug prediction as binary classification tasks. Cami et al. [24] represented drug-drug pairs as feature vectors, and use presence or absence of interactions as labels, and then built logistic regression models. Cheng et al. [25] applied five predictive models (naive Bayes, decision tree, *k*-nearest neighbor, logistic regression, and support vector machine) to build prediction models. Besides similarity-based methods and classification-based methods, there are several methods designed for specific purposes. Takarabe et al. [26] constructed a multi-level drug-drug interaction network, and analyzed, characterized and classified adverse drug-drug interactions. Huang et al. [27] developed a target-center system for each drug, which consists of drug targets and their neighbors in the PPI network and human tissue gene expression.

Since many DDIs are not detected or observed in clinical trials, this work is aimed to predict undetected or unobserved drug-drug interactions. Classification methods utilize two classes of data: annotated drug-drug interaction pairs and annotated non-interaction pairs to build classification models. In the binary classification, known interactions are used as positive instances, but other drug pairs may have undetected or unobserved interactions, which need to be predicted. In machine learning, similar problems are transformed as semi-supervised learning tasks. For this reason, we build DDI prediction models under the frame of semi-supervised learning.

In this paper, we collect drug substructure data, drug target data, drug enzyme data, drug transporter data, drug pathway data, drug indication data, drug side effect data, drug off side effect data and known drug-drug interactions. Multi-source data provide biological information, chemical information, phenotypic information and known interactions to characterize drug-drug interactions. To make use of diverse information, we adopt three representative methods, i.e., the neighbor recommender method [28, 29], the random walk method and the matrix perturbation method [30], to build different prediction models. According to performances of prediction models, we evaluate the usefulness of different information sources for the DDI prediction. The study reveals that DDI network based on known DDIs can provide the important information for DDI prediction. Further, we present flexible frames of integrating different models with suitable ensemble rules, including the weighted average ensemble rule and the classifier ensemble rule, and develop ensemble models to achieve better performances. The experiments demonstrate that ensemble methods can combine diverse information to produce the high-accuracy performances, and outperform existing state-of-the-art methods.

## Methods

### Datasets

The FDA Adverse Event Reporting System (FAERS) is a database which contains adverse event reports and medication error reports submitted to FDA. Tatonetti processed adverse event reports in the AERS, and constructed a database named “TWOSIDES” [31] which contains side effects caused by the combination of drugs. There are 645 drugs and 63,473 distinct pairwise DDIs from unsafe co-prescriptions in TWOSIDES.

The biological information, chemical information and phenotypic information about drugs may be associated with drug-drug interactions. PubChem Compound database [12, 15] can provide drug structures. DrugBank database [10, 11, 16, 17] is a bioinformatics resource with drug targets, drug enzymes and drug transporters. KEGG database [13] is an information resource for protein pathways. Drug targets are mapped to KEGG to obtain drug pathways. SIDER database [14] contains 1430 drugs and 5880 side effect terms which are compiled from public documents and package inserts. Drug side effects and indications are available in SIDER. OFFSIDES database [31] contains 1332 drugs and 10,093 “off-label” side effects.

We map drugs in TWOSIDES to SIDER, OFFSIDES, PubChem and DrugBank. As shown in Table 1, we obtain 548 drugs and 48,584 pairwise DDIs, and substructure data, target data, enzyme data, transporter data, pathway data, indication data, side effect data, off side effect data of these drugs are available. Based on the data, we conduct the comprehensive study to evaluate the usefulness of different data sources for DDI prediction, and discuss how to combine them for the high-accuracy prediction.

**Table 1:** The descriptions about multi-source drug data

Data type	Data	Data Source	Description
Chemical	Substructures	PubChem	881 substructure types
Biological	Targets	DrugBank	780 target types
Biological	Transporters	DrugBank	78 transporter types
Biological	Enzymes	DrugBank	129 enzyme types
Biological	Pathways	KEGG	253 pathway types
Phenotypic	Indications	SIDER	4897 indication types
Phenotypic	Side effects	SIDER	4897 side effect types
Phenotypic	Off side effects	OFFSIDES	9496 off side effects types
Network	Drug-drug interaction network	TWOSIDES	548 drugs and 48,584 DDIs

### DDI prediction based on multi-source data

Multi-source data provide different information for the DDI prediction. Here, we describe how to build models based on different data.

Drug-drug similarities bring important clues for the DDI prediction, and different similarities can be extracted from multi-source data. Drug data are classified as four types, i.e., chemical data, biological data, phenotypic data and the drug-drug interaction network data (formed by known drug-drug interactions). On one hand, we calculate the drug-drug similarities in the biological space, chemical space and phenotypic space, by using drug substructures, drug targets, drug enzymes, drug transporters, drug pathways, drug indications, drug side effects and drug off side effects. On the other hand, we calculate the drug-drug similarities in the drug-drug interaction network. In order to utilize drug-drug similarities, we consider two representative methods [28, 32]: the neighbor recommender method and random walk method, and build DDI prediction models.

We take drugs as nodes and known interactions as edges in the DDI network, and transform the DDI prediction problem as a missing link prediction task. The missing link prediction is an important topic of theoretical interest and practical significance in the complex network [33]. Recently, a novel method named “matrix perturbation method” [30] is proposed, which utilize the network to predict missing links (unobserved DDIs). The studies demonstrated that this method outperforms other missing link prediction methods. Therefore, we adopt the matrix perturbation method to predict potential DDIs based on the DDI network.

In the following context, Similarity-based DDI prediction based on multi-source data presents how to extract different drug-drug similarities from different data and how to develop similarity-based models; Matrix perturbation method for DDI prediction presents the missing link prediction method (matrix perturbation method).

### Similarity-based DDI prediction based on multi-source data

#### Drug-drug similarity based on biological data, chemical data and phenotypic data

A drug can be represented as a binary feature vector, by using drug substructures, drug targets, drug enzymes, drug transporters, drug pathways, drug indications, drug side effects, or drug off side effects. Dimensions of the feature vector respond to presence or absence of components with values 1 or 0. For example, there are 881 types of drug substructures, and a drug can be transformed as an 881-dimensional vector.

Given a drug *x* and a drug *y*, their feature vectors are *V*_*x*_ and *V*_*y*_, and the similarity between *x* and *y* is then calculated by Jaccard formula:

$$ S\left({V}_x,{V}_y\right)=\frac{M_{11}}{M_{01}+{M}_{10}+{M}_{11}} $$

where *M*_11_ is the number of dimensions where *V*_*x*_ and *V*_*y*_ both have a value of 1; *M*_01_ is the number of dimensions where *V*_*x*_ has a value of 0 and *V*_*y*_ has a value of 1; *M*_10_ is the number of dimensions where *V*_*x*_ has a value of 1 and *V*_*y*_ has a value of 0.

Therefore, we can obtain 8 drug feature-based drug-drug similarities, including substructure-based similarity, target-based similarity, enzyme-based similarity, transporter-based similarity, pathway-based similarity, indication-based similarity, side effect-based similarity and off side effect-based similarity.

#### Drug-drug similarity based on known drug-drug interactions

By considering drugs as nodes and interaction as edges, known DDIs can form a DDI network. We calculate drug-drug similarities in the DDI network [33]. The adjacent matrix of the DDI network is denoted as *A* = (*a*_*ij*_), and denotes the set of nodes linked to node. Several similarities between a drug *x* and a drug *y* can be defined.

Common neighbor similarity *S*_*CN*_(*x*, *y*) takes the number of common neighbors between two nodes,

$$ {S}_{CN}\left(x,y\right)=\left|\Gamma (x)\cap \Gamma (y)\right| $$

Adamic-Adar similarity *S*_*AA*_(*x*, *y*) is the counting of common neighbors by assigning the less connected neighbors more weights,

$$ {S}_{AA}\left(x,y\right)={\displaystyle \sum_{z\in \Gamma (x)\cap \Gamma (y)}\frac{1}{ \log \left|\Gamma (z)\right|}} $$

Resource Allocation similarity *S*_*RA*_(*x*, *y*) is based on the complex network resource allocation dynamics,

$$ {S}_{RA}\left(x,y\right)={\displaystyle \sum_{z\in \Gamma (x)\cap \Gamma (y)}\frac{1}{\left|\Gamma (z)\right|}} $$

Katz similarity *S*_*Katz*_(*x*, *y*) sums over the collection of paths with exponential damping according to path lengths,

$$ {S}_{Katz}\left(x,y\right)=\alpha {A}_{xy}+{\alpha}^2{A}_{xy}^2+{\alpha}^3{A}_{xy}^3+\cdots ={\left(I-\alpha A\right)}^{-1}-I $$

where *α* is a parameter, and *I* is the identity matrix. |*α*| < 1/*λ*_max_ is the condition for the compact form, and *λ*_max_ is the largest eigenvalue of *A*.

Average Commute Time similarity *S*_*ACT*_(*x*, *y*) is the average number of steps required by a random walker starting from one node to reach another,

$$ {S}_{ACT}\left(x,y\right)=\frac{1}{l_{xx}^{+}+{l}_{yy}^{+}-2{l}_{xy}^{+}} $$

where *L* ^+^ is the pseudoinverse of the Laplacian matrix for the network.

The random walk with restart similarity *S*_*RWR*_(*x*, *y*) is the probability that a random walker starting from an initial node *x* reaches *y*. The walker moves with the probability *μ* of returning to the initial node and the probability 1 − *μ* going to adjacent nodes,

$$ {S}_{RWR}\left(x,y\right)={q}_{xy}+{q}_{yx} $$

where *q* = (1 − *μ*)(1 − *μP* ^*T*^)^− 1^ *A*, and *P* = *D* ^− 1^ *A* is the normalized transition matrix of the adjacency matrix *A*, and *D* is the degree matrix of *A*.

Therefore, we obtain 6 DDI network-based drug-drug similarities, including common neighbor similarity, Adamic-Adar similarity, resource allocation similarity, Katz similarity, average commute time similarity and random walk with restart similarity.

#### Similarity-based methods for DDI prediction

Given a *N* × *N* similarity matrix *S* = (*s*_*ij*_) for *N* drugs, known pairwise DDIs are denoted by an adjacent matrix *A* = (*a*_*ij*_). The neighbor recommender method and the random walk method are briefly introduced as follows.

The neighbor recommender method [28, 34] is one of most popular methods in recommender systems, which recommends items (movies, music, books, et al.) to users, or predicts the ‘rating’ or ‘preference’ that users would give to items. The neighbor recommender method takes the weighted average information of neighbors for prediction. *Y*_*ij*_ = ∑_*k* = 1,*k* ≠ *j*_^*N*^ *s*_*ik*_ *a*_*kj*_/∑_*k* = 1,*k* ≠ *j*_^*N*^ *s*_*ik*_ is calculated for drug_*i*_ and drug_*j*_ which don’t have known interaction, where *s*_*ik*_ is the similarity between drug_*i*_ and drug_*k*_, and *a*_*kj*_ = 1 *or* 0 means interaction or non-interaction between drug_*k*_ and drug_*j*_. We can calculate *Y*_*ji*_ in this same way. The probability that drug_*i*_ interacts with drug_*j*_ *score*_*ji*_ = *score*_*ij*_ = *Y*_*ij*_ + *Y*_*ji*_.

A random walk is a mathematical formalization of a path that consists of a succession of random steps. There are a great number of successful applications in the network analysis [35–38]. In random walk, a random walker starts from an initial node, and moves to neighbors with the probability *μ* and moves back to the initial node with the probability 1 − *μ*. The similarity matrix *S* is normalized as *W* = *D* ^− 1^ *S*, where *D* is the degree matrix of *S*. The matrix form of the update is summarized as *Y* = *μWY* + (1 − *μ*)*A*, and it will converge to the solution: *Y* = (1 − *μ*)(*I* − *μW*)^− 1^ *A*. The probability that drug_*i*_ interacts with drug_*j*_ *score*_*ji*_ = *score*_*ij*_ = *Y*_*ij*_ + *Y*_*ji*_.

### Matrix perturbation method for DDI prediction

The matrix perturbation method assumes that random removal of a small proportion of links from a network will not change the network structure [30], which is reflected by eigenvectors of its adjacent matrix.

Let’s introduce notations for the matrix perturbation method. Given the drug-drug interaction network *G*(*V*, *E*), *V* is the set of nodes, and *E* is the set of edges. The adjacent matrix is *A* = (*a*_*ij*_), and the eigenvectors and eigenvalues of the adjacent matrix are denoted by *x*_*k*_ and *λ*_*k*_, *k* = 1, 2, ⋯, *N*.

A fraction of links Δ*E* are randomly removed from *E*, and the set of remaining links *E* ^*R*^ = *E* − Δ*E*. Thus, we obtain the new network *G* ^*R*^(*V*, *E* ^*R*^) with the adjacent matrix *A* ^*R*^ = *A* − Δ*A*, where Δ*A* is the adjacent matrix for removed links. Then, we calculate the eigenvectors *x*_*k*_^*R*^ and eigenvalues *λ*_*k*_^*R*^ of *A* ^*R*^, *k* = 1, 2, ⋯, *N*. We denote that *A* = *A* ^*R*^ + Δ*A*, *x*_*k*_ = *x*_*k*_^*R*^ + Δ*x*_*k*_ and *λ*_*k*_ = *λ*_*k*_^*R*^ + Δ*λ*_*k*_.

In the network *G*(*V*, *E*), the relation of eigenvectors, eigenvalues and the adjacent matrix is written as,

$$ \left({A}^R+\Delta A\right)\left({x}_k^R+\Delta {x}_k\right)=\left({\lambda}_k^R+\Delta {\lambda}_k\right)\left({x}_k^R+\Delta {x}_k\right) $$

By left multiplying (*x*_*k*_^*R*^)^*T*^ in above equation, we can obtain $\Delta {\lambda}_k\approx \frac{{\left({x}_k^R\right)}^T\Delta A{x}_k^R}{{\left({x}_k^R\right)}^T{x}_k^R}$.

We estimate eigenvalues *λ*_*k*_ = *λ*_*k*_^*R*^ + Δ*λ*_*k*_, and keep eigenvectors *x*_*k*_^*R*^ unchanged. Then, we reconstruct the adjacent matrix of *G*(*V*, *E*) by summing eigenvalues and eigenvectors,

$$ \tilde{A}={\displaystyle \sum_{i=1}^N\left({\lambda}_k^R+\Delta {\lambda}_k\right)}{x}_k^R{\left({x}_k^R\right)}^T $$

The probability that drug_*i*_ interacts with drug_*j*_ *score*_*ij*_ = *score*_*ji*_ = *Ã*_*ij*_ + *Ã*_*ji*_. More details are available in the publication [30].

### Combining multi-source data for DDI prediction

Since we build different prediction models based on different data, it is natural to combine them for better performance. Ensemble learning is a useful technique that aggregates multiple machine learning models to achieve overall high prediction accuracy as well as good generalization [39]. Ensemble learning has been applied to a great number of applications in bioinformatics [29, 40, 41].

**Fig. 1:**
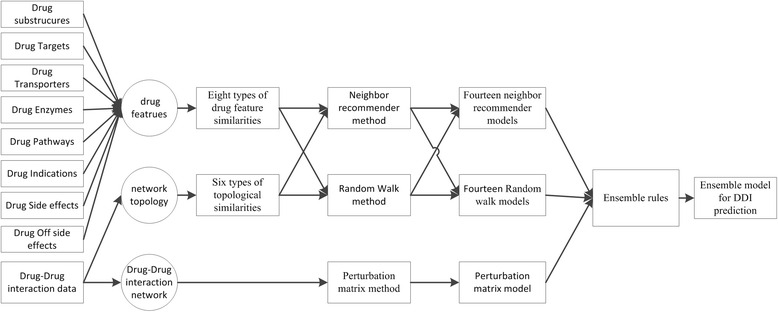
The scheme of integrating multi-source data for DDI prediction

An ensemble learning system usually has two components: base predictors and ensemble rules. In our ensemble system, we adopt heterogeneous models {*f*_*i*_}_*i* = 1_^*n*^ based on multi-source data as base predictors. To integrate base predictors, we consider two popular ensemble rules: the weighted average ensemble rule and the classifier ensemble rule. Figure 1 demonstrates the flowchart of ensemble systems.

The weighted average ensemble rule takes the weighted average of outputs from base predictor. For a new input *x*_*new*_, base predictors give out the predictions {*f*_*i*_(*x*_*new*_)}_*i* = 1_^*n*^, and their weighted average ∑_*i* = 1_^*n*^ *w*_*i*_ *f*_*i*_(*x*_*new*_) is adopted as the prediction of the ensemble model, where ∑_*i* = 1_^*n*^ *w*_*i*_ = 1 and *w*_*i*_ ≥ 0. We adopt the genetic algorithm (GA) to determine weights in the ensemble model. In the GA optimization, candidate weights are represented as chromosomes, and the fitness of a chromosome is the area under the precision-recall curve (AUPR) score of the ensemble model on the validation data. The objective function of GA optimization is to maximize the AUPR score.

The classifier ensemble rule is to seek a classification function *G* : (*f*_1_(*x*), *f*_2_(*x*), ⋯, *f*_*n*_(*x*)) → {0, 1}, which maps outputs of *n* base predictors to a label. For a new input *x*_*new*_, outputs of base predictors are {*f*_*i*_(*x*_*new*_)}_*i* = 1_^*n*^, and the prediction of the classifier ensemble model is *G*(*f*_1_(*x*_*new*_), *f*_2_(*x*_*new*_), ⋯, *f*_*n*_(*x*_*new*_)). Here, we adopt logistic regression as the classification function.

## Results and discussion

### Evaluation metrics

We adopt *k*-fold cross validation (*k*-CV) to evaluate prediction models. Known interactions are randomly split into *k* subsets with equal size. In each fold, one subset is used as the testing set; 80 and 20% of other interactions (*k*-1 subsets) are used as the training set and validation set. Base predictors are constructed on the training set, and parameters in the ensemble system are tuned by using the validation set. Then, the ensemble model makes predictions for the testing set. This procedure is repeated until each subset is ever used for testing. To avoid the bias of data split, we implement 20 independent runs of *k*-CV for each model, and average performances are adopted.

Here, we adopt several evaluation metrics to measure performances of prediction models, i.e., accuracy (ACC), precision, recall, F-measure (F), area under ROC curve (AUC) and the area under the precision-recall curve (AUPR). In our task, DDIs take a small proportion of all drug pairs, and thus AUPR, which takes into account both recall and precision, is used as the primary evaluation metric.

### Performances of different models based on multi-source data

We extract 14 different similarities from multi-source data, and respectively adopt the neighbor recommender method and the random walk method to build 28 similarity-based prediction models. By formulating the original problem as a missing link prediction task, we adopt the matrix perturbation method to build the prediction model based on known DDIs. Therefore, we construct 29 prediction models based on multi-source data. Since different models utilize different information for DDI prediction, performances of the models are indicators for the usefulness of information sources.

As shown in Table 2, these models produce different performances on the benchmark dataset in the cross validation. Among eight feature-based similarities, substructure similarity, side effect similarity, off side effect similarity and indication similarity lead to better performances than other similarities, indicating that drug substructures, drug side effects, drug off side effects and drug indications provide important information for the drug-drug interactions. Among the network topology-based similarities, RA and RWR can produce better results. The comparison shows that drug feature-based similarities as well as topological similarities can provide useful information to characterize drug-drug interactions and lead to useful models. The matrix perturbation method utilizes the DDI network as a whole to make predictions. Among all prediction models, the matrix perturbation method produces the best results, indicating that known DDIs provide one of most useful information to identify potential DDIs.

**Table 2:** Performances of different models evaluated by 20 runs of 5-CV

Method	Similarity	Index	AUC	AUPR	Recall	Precision	Accuracy	F
Neighbor recommender Method	Substructure	1	0.936	0.759	0.765	0.617	0.950	0.683
	Target	2	0.820	0.365	0.338	0.548	0.867	0.418
	Transporter	3	0.714	0.329	0.290	0.389	0.862	0.331
	Enzyme	4	0.756	0.377	0.471	0.346	0.909	0.399
	Pathway	5	0.812	0.571	0.657	0.474	0.932	0.550
	Indication	6	0.912	0.599	0.555	0.591	0.923	0.572
	Label	7	0.936	0.754	0.750	0.618	0.949	0.678
	Off label	8	0.940	0.768	0.765	0.629	0.951	0.691
	CN	9	0.941	0.767	0.745	0.635	0.949	0.685
	AA	10	0.941	0.767	0.747	0.634	0.949	0.686
	RA	11	0.943	0.770	0.752	0.634	0.950	0.688
	Katz	12	0.937	0.735	0.707	0.608	0.944	0.653
	ACT	13	0.931	0.752	0.723	0.618	0.947	0.667
	RWR	14	0.941	0.766	0.746	0.634	0.949	0.685
Random walk Method	Substructure	15	0.936	0.758	0.763	0.616	0.950	0.681
	Target	16	0.852	0.559	0.596	0.501	0.927	0.544
	Transporter	17	0.713	0.363	0.297	0.381	0.864	0.329
	Enzyme	18	0.760	0.470	0.657	0.344	0.927	0.451
	Pathway	19	0.811	0.594	0.709	0.479	0.937	0.572
	Indication	20	0.941	0.777	0.768	0.641	0.952	0.699
	Label	21	0.936	0.760	0.764	0.621	0.950	0.685
	Off label	22	0.937	0.763	0.761	0.627	0.950	0.688
	CN	23	0.938	0.757	0.736	0.625	0.948	0.676
	AA	24	0.938	0.755	0.734	0.624	0.947	0.675
	RA	25	0.937	0.748	0.729	0.616	0.946	0.667
	Katz	26	0.937	0.750	0.730	0.619	0.946	0.669
	ACT	27	0.930	0.748	0.727	0.632	0.938	0.671
	RWR	28	0.939	0.764	0.742	0.635	0.949	0.684
Matrix perturbation method		29	0.948	0.782	0.755	0.666	0.952	0.707

We also conduct 20 runs of 3-CV to evaluate prediction models, and results are shown in Table 3. The comparison between 3-CV results and 5-CV results demonstrates that prediction models have different performances under different experimental conditions, and a model cannot produce the best results in all cases. For example, the matrix perturbation method assumes that the topology of a network will not change if only a small proportion of links are removed. In 3-CV, more links are kept for testing, and the predictive power may be affected. Therefore, the matrix perturbation method is not the best predictor in 3-CV experiments. For this reason, we integrate different models to make robust predictions.

**Table 3:** Performances of different models evaluated by 20 runs of 3-CV

Method	Similarity	Index	AUC	AUPR	Recall	Precision	Accuracy	F
Neighbor recommender Method	Substructure	1	0.935	0.808	0.772	0.669	0.927	0.717
	Target	2	0.806	0.425	0.420	0.579	0.831	0.486
	Transporter	3	0.714	0.405	0.344	0.495	0.800	0.406
	Enzyme	4	0.753	0.437	0.466	0.424	0.853	0.443
	Pathway	5	0.810	0.624	0.674	0.510	0.898	0.581
	Indication	6	0.903	0.640	0.584	0.658	0.888	0.618
	Label	7	0.935	0.803	0.758	0.673	0.925	0.713
	Off label	8	0.939	0.815	0.771	0.684	0.928	0.725
	CN	9	0.940	0.816	0.761	0.691	0.927	0.724
	AA	10	0.941	0.816	0.761	0.690	0.927	0.724
	RA	11	0.942	0.819	0.763	0.691	0.928	0.725
	Katz	12	0.933	0.782	0.715	0.666	0.917	0.689
	ACT	13	0.866	0.721	0.629	0.574	0.915	0.600
	RWR	14	0.940	0.814	0.760	0.688	0.927	0.722
Random walk Method	Substructure	15	0.935	0.807	0.768	0.670	0.927	0.716
	Target	16	0.844	0.608	0.601	0.555	0.888	0.576
	Transporter	17	0.713	0.437	0.339	0.504	0.795	0.404
	Enzyme	18	0.760	0.533	0.655	0.374	0.886	0.476
	Pathway	19	0.810	0.648	0.724	0.515	0.906	0.601
	Indication	20	0.939	0.820	0.773	0.693	0.930	0.731
	Label	21	0.936	0.809	0.771	0.674	0.927	0.719
	Off label	22	0.937	0.811	0.771	0.680	0.928	0.722
	CN	23	0.937	0.807	0.748	0.685	0.925	0.715
	AA	24	0.937	0.806	0.747	0.683	0.924	0.714
	RA	25	0.936	0.799	0.741	0.675	0.923	0.706
	Katz	26	0.936	0.801	0.743	0.677	0.923	0.708
	ACT	27	0.866	0.706	0.658	0.699	0.834	0.643
	RWR	28	0.938	0.813	0.759	0.690	0.927	0.723
Matrix perturbation method		29	0.941	0.813	0.755	0.709	0.928	0.731

### Performances of ensemble models

Based on multi-source data, we construct 29 prediction models including 28 similarity-based models and one perturbation matrix model. We use these models as base predictors, and respectively adopt the weighted average ensemble rule and the classifier ensemble rule to build ensemble models.

We apply the genetic algorithm (GA) to determine optimal weights in the weighted average ensemble models. GA is implemented by using python package “deap”. The initial population has 100 chromosomes. In the population update, the elitist strategy is used for the selection operator, and default parameters are adopted for the mutation probability and crossover probability. The population update terminates when the change of best fitness scores is less than the default value of 1E-6 or the max generation number of 50 is reached.

To build classifier ensemble models, we train the logistic regression classifier to combine outputs of base predictors. The logistic regression is implemented by using python package “scikit-learn”. Default parameters are used; L1 regularization and L2 regularization are respectively considered. In the following context, classifier ensembles models refer to logistic regression ensemble models.

Table 4 shows 3-CV results and 5-CV results. In 5-CV experiments, the weighted average ensemble model, the classifier ensemble model (L1 regularization) and classifier ensemble model (L2 regularization) produce the AUPR scores of 0.795, 0.807 and 0.806; in 3-CV experiments, three models yield the AUPR scores of 0.832, 0.841 and 0.839. The comparison demonstrates that the classifier ensemble models produce better results than the weighted average ensemble model. The possible reason is that the weighted average ensemble method uses the linear function for ensemble learning and classifier ensemble method trains nonlinear function. Moreover, the classifier ensemble method with L1 regularization can produce better results than the classifier ensemble method with L2 regularization, for L1 regularization can produce the sparse model and enhance the generalization capability.

**Table 4:** Performances of ensemble model evaluated by 20 runs of 3-CV and 5-CV

Evluation	Method	AUC	AUPR	Precision	Recall	Accuracy	F-measure
3-CV evaluation	Weighted average ensemble method	0.947	0.832	0.782	0.703	0.932	0.740
	Classifier ensemble method(L1)	0.954	0.841	0.788	0.717	0.934	0.751
	Classifier ensemble method(L2)	0.952	0.839	0.784	0.712	0.933	0.746
5-CV evaluation	Weighted average ensemble method	0.951	0.795	0.775	0.659	0.953	0.712
	Classifier ensemble method(L1)	0.957	0.807	0.785	0.670	0.955	0.723
	Classifier ensemble method(L2)	0.956	0.806	0.783	0.665	0.955	0.719

Clearly, ensemble models produce better results than base predictors. In 5-CV experiments, the classifier ensemble method (L1) can improve the AUPR score of 0.782 (produced by the matrix perturbation model) to 0.806. Since we implement 20 runs of 5-CV for ensemble models and matrix perturbation models, we conduct *t*-test to test the difference of their performances in terms of AUPR score, and the statistical significance is observed (*p*-value =1.21E-39). In 3-CV experiments, the classifier ensemble method (L1) can enhance the AUPR score from 0.820 (produced by the indication-based random walk model) to 0.839, and we also observe the statistical significance of improvement between the classifier ensemble model (L1) and the indication-based random walk model (*p*-value =3.12E-41).

**Fig. 2:**
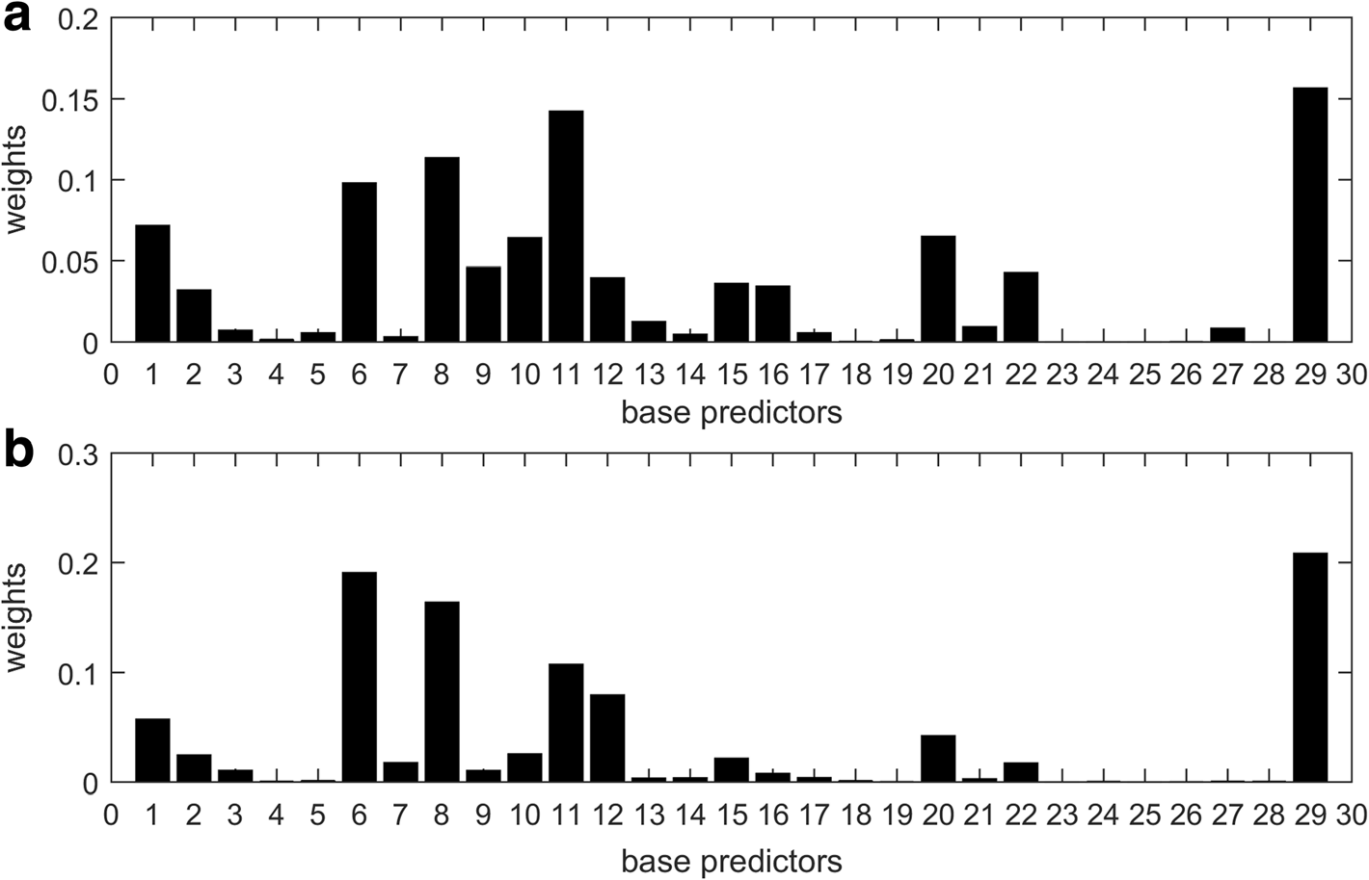
Weights for base predictors in the weighted average ensemble models (**a**) 3-CV experiments; (**b**) 5-CV experiments

Further, we investigate into details of the ensemble models based on 3-CV results and 5-CV results. Firstly, we analyze weights in the weighted average ensemble models determined by GA. There are 100 sets of weights for 20 runs of 5-CV; there are 60 sets of weights for 20 runs of 3-CV. We calculate the average weights for each predictor, and visualize the normalized weights in Fig. 2. Base predictors with high AURP scores may be assigned great weights. For example, the matrix perturbation model produces best 5-CV results, and thus gains the greatest weight in the ensemble models. We observe that several base predictors (such as RWR-based random walk model) are not used in the ensemble models. The classifier ensemble method (L1) produces the sparse models, which integrate the subset of base predictors. According to 5-CV results, several base predictors (index: 1, 10, 15, 21, 22, 27, 28, 29) are not used in the classifier ensemble model. In the view of computer science, multi-source data provide diverse information but also bring the redundant information. Combining base predictors is a combinatorial optimization problem. Therefore, the weighted average ensemble method and the classifier ensemble method (L1) use a subset of base predictors to develop ensemble models.

### Comparison with existing state-of-the-art methods

Since this work is designed to predict undetected or unobserved DDIs, we adopt methods of the same type for comparison. Vilar used known interactions of most similar drugs to predict DDIs, and proposed the substructure similarity-based model [19] and interaction profile fingerprint (also known as common neighbors, CN) similarity-based model [20]. Zhang [23] adopted the label propagation algorithm to build substructure similarity-based model, side effect similarity-based model and off side effect similarity-based model. We name these models as Vilar’s substructure-based model, Vilar’s CN index-based model, substructure-based label propagation model, side effect-based label propagation model and off side effect-based label propagation model. These prediction models are implemented according to details in publications. All models are evaluated by 20 runs of cross validation under the same conditions.

As shown in Table 5, our ensemble methods produce better results than other state-of-the-art methods in terms of different metrics. The classifier ensemble method (L1) produces the best results in both 3-CV experiments and 5-CV experiments. Further, we adopt *t*-test to compare the ensemble methods with other state-of-the-art methods in terms of AUPR scores. Table 6 demonstrates that our ensemble methods produce significantly better results (*p* < 0.05 in terms of AUPR scores).

**Table 5:** Performances of the ensemble method and benchmark methods evaluated by 20 runs of 3-CV and 5-CV

Evluation	Method	AUC	AUPR	Precision	Recall	Accuracy	F-measure
3-CV evaluation	Vilar’s substructure-based model	0.670	0.273	0.145	0.535	0.684	0.229
	Vilar’s CN index-based model	0.872	0.413	0.377	0.553	0.880	0.447
	Substructure-based label propagation model	0.935	0.807	0.768	0.670	0.927	0.716
	Side effect-based Label propagation model	0.936	0.809	0.771	0.674	0.927	0.719
	Off side effect-based label propagation model	0.937	0.811	0.771	0.680	0.928	0.722
	Weighted average ensemble method	0.947	0.832	0.782	0.703	0.932	0.740
	Classifier ensemble method (L1)	0.954	0.841	0.788	0.717	0.934	0.751
	Classifier ensemble method (L2)	0.952	0.839	0.784	0.712	0.933	0.746
5-CV evaluation	Vilar’s substructure-based model	0.670	0.273	0.145	0.535	0.684	0.229
	Vilar’s CN index-based model	0.872	0.413	0.377	0.553	0.880	0.447
	Substructure-based label propagation model	0.936	0.758	0.763	0.616	0.950	0.681
	Side effect-based Label propagation model	0.936	0.760	0.764	0.621	0.950	0.685
	Off side effect-based label propagation model	0.937	0.763	0.761	0.627	0.950	0.688
	Weighted average ensemble method	0.951	0.795	0.775	0.659	0.953	0.712
	Classifier ensemble method (L1)	0.957	0.807	0.785	0.670	0.955	0.723
	Classifier ensemble method (L2)	0.956	0.806	0.783	0.665	0.955	0.719

**Table 6:** The statistical significance of performance improvements achieved by our ensemble methods

Evaluation	Methods	Weighted average ensemble method	Classifier ensemble method(L1)	Classifier ensemble method(L2)
3-CV	Vilar’s substructure-based model	1.05E-94	2.67E-78	1.18E-86
	Vilar’s CN index-based model	4.12E-74	7.32E-67	1.14E-71
	Substructure-based label propagation model	1.02E-45	8.30E-34	2.96E-41
	Side effect-based Label propagation model	1.61E-44	8.86E-33	3.28E-40
	Off side effect-based label propagation model	3.32E-42	1.94E-31	1.17E-38
5-CV	Vilar’s substructure-based model	4.76E-52	3.12E-48	5.42E-54
	Vilar’s CN index-based model	2.27E-48	2.34E-44	1.14E-48
	Substructure-based label propagation model	1.68E-31	1.71E-29	1.28E-36
	Side effect-based Label propagation model	1.27E-30	6.71E-29	3.04E-36
	Off side effect-based label propagation model	4.03E-30	2.43E-28	1.67E-35

**Fig. 3:**
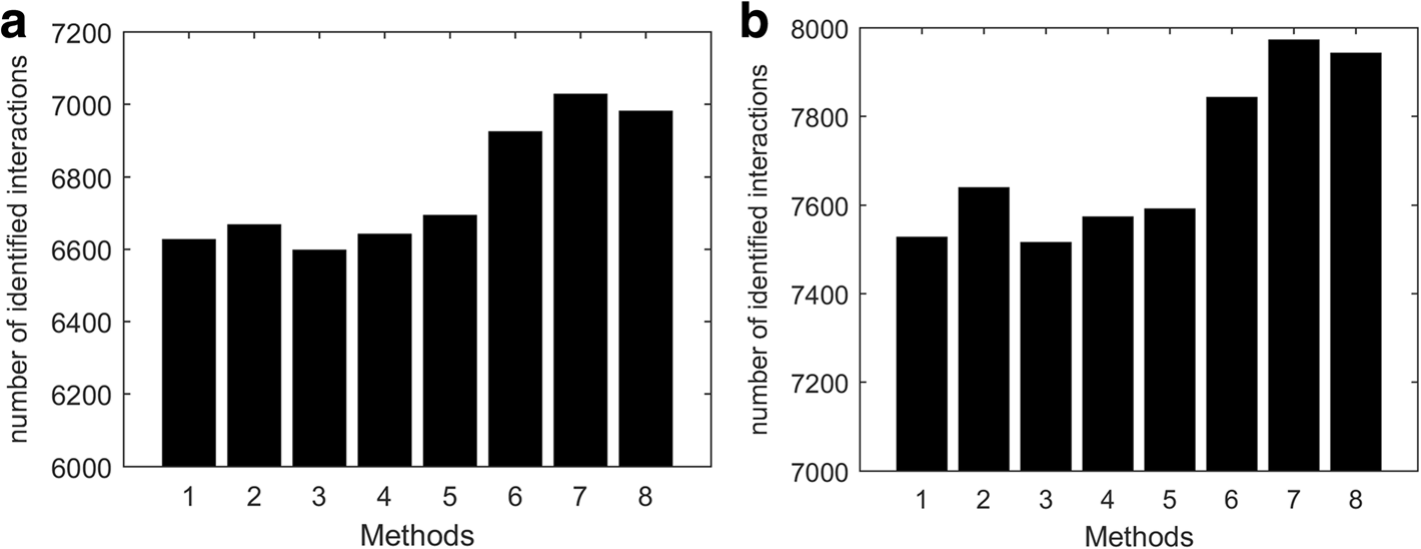
The number of identified testing interactions (**a**) top 10,000 predictions; (**b**) top 15, 000 predictions. 1: Vilar’s substructure-based model (6626, 7527); 2: Vilar’s CN index-based model (6667, 7639); 3: Substructure-based label propagation model (6597, 7515); 4: Side effect-based Label propagation model (6641, 7573); 5: Off side effect-based label propagation model (6693,7591); 6: Weighted average ensemble method (6923, 7842); 7: L1 Classifier ensemble method (7027, 7972); 8: L2 Classifier ensemble method (6980, 7942)

In one fold of 5-fold cross validation, we adopt 80% interactions (38,868) as the training set and the validations set, and use other interactions (9716) as the testing set. We build the prediction model based on the training set and the validations set, and then make predictions for non-interaction drug-drug pairs (111,010) to identify testing interactions (9716). Based on the result, we respectively count how many testing DDIs are identified in the top 10,000 predictions and top 15,000 predictions. As shown in Fig. 3, the classifier ensemble model (L1) can identify 7027 testing interactions when verifying top 10,000 predictions, and identify 7842 testing interactions when verifying top 15,000 predictions. In general, our ensemble models can identify 300 ~ 400 more interactions than other methods do.

### Predicted novel interactions

In this paper, we use the benchmark dataset with 548 drugs and 48,584 pairwise drug-drug interactions from TWOSIDES database. There are 149,878 drug-drug pairs between these drugs. Besides 48,584 known pairwise DDIs, 101294 remaining drug pairs (“non-interaction pairs”) may contain undetected or unobserved DDIs, which are not available in TWOSIDES. We train the prediction models based on 548 drugs and 48,584 known DDIs, and predict unobserved DDIs. In the prediction, great scores of drug pairs indicate high probabilities of having interactions, and the prediction results are transformed as a recommendation list of unobserved interactions or novel interactions. To confirm novel interactions, we look up them in the latest online version of DrugBank database. Table 7 lists top 20 novel interactions predicted by our method, and a significant fraction of novel interactions (7 out of 20) are confirmed in DrugBank database.

**Table 7:** Top 20 novel interactions predicted by our method (confirmed interactions shown in bold)

Rank	ID1	ID2	Drug name 1	Drug name 2
1	**DB00495**	**DB00451**	**Zidovudine**	**Levothyroxine**
2	**DB01193**	**DB00264**	**Acebutolol**	**Metoprolol**
3	DB00945	DB01033	Acetylsalicylic acid	Mercaptopurine
4	DB01059	DB00448	Norfloxacin	Lansoprazole
5	DB00990	DB00635	Exemestane	Prednisone
6	DB00213	DB00310	Pantoprazole	Chlorthalidone
7	**DB01197**	**DB00468**	**Captopril**	**Quinine**
8	DB00658	DB00331	Sevelamer	Metformin
9	**DB00571**	**DB01203**	**Propranolol**	**Nadolol**
10	DB00537	DB00869	Ciprofloxacin	Dorzolamide
11	**DB01264**	**DB00863**	**Darunavir**	**Ranitidine**
12	DB00346	DB00630	Alfuzosin	Alendronic acid
13	DB00535	DB00813	Cefdinir	Fentanyl
14	DB00334	DB00795	Olanzapine	Sulfasalazine
15	DB00749	DB01142	Etodolac	Doxepin
16	**DB00820**	**DB01020**	**Tadalafil**	**Isosorbide Mononitrate**
17	DB00472	DB00214	Fluoxetine	Torasemide
18	DB00862	DB00407	Vardenafil	Ardeparin
19	DB00275	DB00959	Olmesartan	Methylprednisolone
20	**DB00540**	**DB00967**	**Nortriptyline**	**Desloratadine**

Further, we compare the ensemble model and the matrix perturbation model by testing their capability of finding out novel interactions. The top 1000 novel interactions predicted by the ensemble model and the matrix perturbation model are provided in supplementary material (see Additional file 1). For each method, we find evidences in DrugBank to confirm novel interactions. If we look up all 1000 interactions of the matrix perturbation model and the ensemble model, we can confirm 297 novel interactions and 318 novel interactions respectively (252 common interactions are shared). Further, based on the top 1000 novel interactions, we use the number of predictions as X-axis and the number of confirmed novel interactions in the predictions as Y-axis, and then visualize performances of two models (see Additional file 2). In general, the ensemble model can find out more novel interactions than the matrix perturbation model, indicating the usefulness of integrating multi-source data.

## Conclusions

The prediction of drug-drug interactions is an important task in the drug discovery, which helps to reduce potential risks and understand the mechanism of drug-drug interactions. This paper collects a wide variety of drug data, and designs the models based on multi-source data for the DDI prediction. Compared with existing DDI prediction methods, our methods produce better performances, and the statistical analysis demonstrates that the performance improvements achieved by our method are statistically significant. In conclusion, the proposed methods are promising for the DDI prediction.

## Additional files

Additional file 1: Top 1000 novel interactions predicted by the ensemble model and the matrix perturbation model. (XLSX 75 kb)
Additional file 2: Visualization of the number of predictions vs. number of confirmed interactions. (TIF 519 kb)

## Abbreviations

5-CV: 5-fold cross validation
AUC: Area under ROC curve
AUPR: Area under precision-recall curve
DDI: Drug-drug interaction
GA: Genetic algorithm

## References

[1] Nagai N (2010). Drug interaction studies on new drug applications: current situations and regulatory views in Japan. Drug Metab Pharmacokin.

[2] Percha B, Altman RB (2013). Informatics confronts drug-drug interactions. Trends Pharmacol Sci.

[3] Prueksaritanont T, Chu X, Gibson C, Cui D, Yee KL, Ballard J, Cabalu T, Hochman J (2013). Drug-drug interaction studies: regulatory guidance and an industry perspective. AAPS J.

[4] Kusuhara H (2014). How far should we go? Perspective of drug-drug interaction studies in drug development. Drug Metab Pharmacokin.

[5] Noren GN, Sundberg R, Bate A, Edwards IR (2008). A statistical methodology for drug-drug interaction surveillance. Stat Med.

[6] Tatonetti NP, Denny J, Murphy S, Fernald G, Krishnan G, Castro V, Yue P, Tsau P, Kohane I, Roden D (2011). Detecting drug interactions from adverse‐event reports: interaction between paroxetine and pravastatin increases blood glucose levels. Clin Pharmacol Ther.

[7] Duke JD, Han X, Wang Z, Subhadarshini A, Karnik SD, Li X, Hall SD, Jin Y, Callaghan JT, Overhage MJ (2012). Literature based drug interaction prediction with clinical assessment using electronic medical records: novel myopathy associated drug interactions. PLoS Comput Biol.

[8] Tatonetti NP, Fernald GH, Altman RB (2012). A novel signal detection algorithm for identifying hidden drug-drug interactions in adverse event reports. J Am Med Inform Assoc.

[9] He L, Yang Z, Zhao Z, Lin H, Li Y (2013). Extracting drug-drug interaction from the biomedical literature using a stacked generalization-based approach.

[10] Wishart DS, Knox C, Guo AC, Shrivastava S, Hassanali M, Stothard P, Chang Z, Woolsey J (2006). DrugBank: a comprehensive resource for in silico drug discovery and exploration. Nucleic Acids Res.

[11] Wishart DS, Knox C, Guo AC, Cheng D, Shrivastava S, Tzur D, Gautam B, Hassanali M (2008). DrugBank: a knowledgebase for drugs, drug actions and drug targets. Nucleic Acids Res.

[12] Wang Y, Xiao J, Suzek TO, Zhang J, Wang J, Bryant SH (2009). PubChem: a public information system for analyzing bioactivities of small molecules. Nucleic Acids Res.

[13] Kanehisa M, Goto S, Furumichi M, Tanabe M, Hirakawa M (2010). KEGG for representation and analysis of molecular networks involving diseases and drugs. Nucleic Acids Res.

[14] Kuhn M, Campillos M, Letunic I, Jensen LJ, Bork P (2010). A side effect resource to capture phenotypic effects of drugs. Mol Syst Biol.

[15] Li Q, Cheng T, Wang Y, Bryant SH (2010). PubChem as a public resource for drug discovery. Drug Discov Today.

[16] Knox C, Law V, Jewison T, Liu P, Ly S, Frolkis A, Pon A, Banco K, Mak C, Neveu V (2011). DrugBank 3.0: a comprehensive resource for ‘omics’ research on drugs. Nucleic Acids Res.

[17] Law V, Knox C, Djoumbou Y, Jewison T, Guo AC, Liu Y, Maciejewski A, Arndt D, Wilson M, Neveu V (2014). DrugBank 4.0: shedding new light on drug metabolism. Nucleic Acids Res.

[18] Gottlieb A, Stein GY, Oron Y, Ruppin E, Sharan R (2012). INDI: a computational framework for inferring drug interactions and their associated recommendations. Mol Syst Biol.

[19] Vilar S, Harpaz R, Uriarte E, Santana L, Rabadan R, Friedman C (2012). Drug-drug interaction through molecular structure similarity analysis. J Am Med Inform Assoc.

[20] Vilar S, Uriarte E, Santana L, Tatonetti NP, Friedman C (2013). Detection of drug-drug interactions by modeling interaction profile fingerprints. PLoS One.

[21] Li P, Huang C, Fu Y, Wang J, Wu Z, Ru J, Zheng C, Guo Z, Chen X, Zhou W (2015). Large-scale exploration and analysis of drug combinations. Bioinformatics.

[22] Park K, Kim D, Ha S, Lee D (2015). Predicting pharmacodynamic drug-drug interactions through signaling propagation interference on protein-protein interaction networks. PLoS One.

[23] Zhang P, Wang F, Hu J, Sorrentino R (2015). Label propagation prediction of drug-drug interactions based on clinical side effects. Sci Rep.

[24] Cami A, Manzi S, Arnold A, Reis BY (2013). Pharmacointeraction network models predict unknown drug-drug interactions. PLoS One.

[25] Cheng F, Zhao Z (2014). Machine learning-based prediction of drug-drug interactions by integrating drug phenotypic, therapeutic, chemical, and genomic properties. J Am Med Inform Assoc.

[26] Takarabe M, Shigemizu D, Kotera M, Goto S, Kanehisa M (2011). Network-based analysis and characterization of adverse drug-drug interactions. J Chem Inf Model.

[27] Huang J, Niu C, Green CD, Yang L, Mei H, Han JD (2013). Systematic prediction of pharmacodynamic drug-drug interactions through protein-protein-interaction network. PLoS Comput Biol.

[28] Bobadilla J, Ortega F, Hernando A, Gutiérrez A (2013). Recommender systems survey. Knowl-Based Syst.

[29] Zhang W, Zou H, Luo L, Liu Q, Wu W, Xiao W (2016). Predicting potential side effects of drugs by recommender methods and ensemble learning. Neurocomputing.

[30] Lu L, Pan L, Zhou T, Zhang YC, Stanley HE (2015). Toward link predictability of complex networks. Proc Natl Acad Sci U S A.

[31] Tatonetti NP, Ye PP, Daneshjou R, Altman RB (2012). Data-driven prediction of drug effects and interactions. Sci Transl Med.

[32] Schafer JB, Konstan J, Riedl J. Recommender systems in e-commerce. In: Proceedings of the 1st ACM conference on Electronic commerce. New York: ACM; 1999. p. 158–66.

[33] Lü L, Zhou T (2011). Link prediction in complex networks: a survey. Physica A.

[34] Koren Y, Bell R. Advances in collaborative filtering. In: Recommender Systems Handbook. New York: Springer; 2015. p. 77–118.

[35] Liu W, Lü L (2010). Link prediction based on local random walk. EPL (Europhysics Letters).

[36] Backstrom L, Leskovec J. Supervised random walks: predicting and recommending links in social networks. In: Proceedings of the fourth ACM international conference on Web search and data mining. New York: ACM; 2011. p. 635–44.

[37] Chen X, Liu MX, Yan GY (2012). Drug-target interaction prediction by random walk on the heterogeneous network. Mol BioSyst.

[38] Seal A, Ahn YY, Wild DJ (2015). Optimizing drug-target interaction prediction based on random walk on heterogeneous networks. J Cheminf.

[39] Polikar R (2006). Ensemble based systems in decision making. IEEE Circuits Syst Mag.

[40] Zhang W, Niu Y, Xiong Y, Zhao M, Yu R, Liu J (2012). Computational prediction of conformational B-cell epitopes from antigen primary structures by ensemble learning. PLoS One.

[41] Zhang W, Liu F, Luo L, Zhang J (2015). Predicting drug side effects by multi-label learning and ensemble learning. BMC Bioinf.

