# Supplementary figures and images for: Predicting potential drug-drug interactions by integrating chemical, biological, phenotypic and network data
Source: BMC Bioinformatics. 2017 Jan 5;18:18. doi: 10.1186/s12859-016-1415-9 (PMC5217341; doi:10.1186/s12859-016-1415-9)

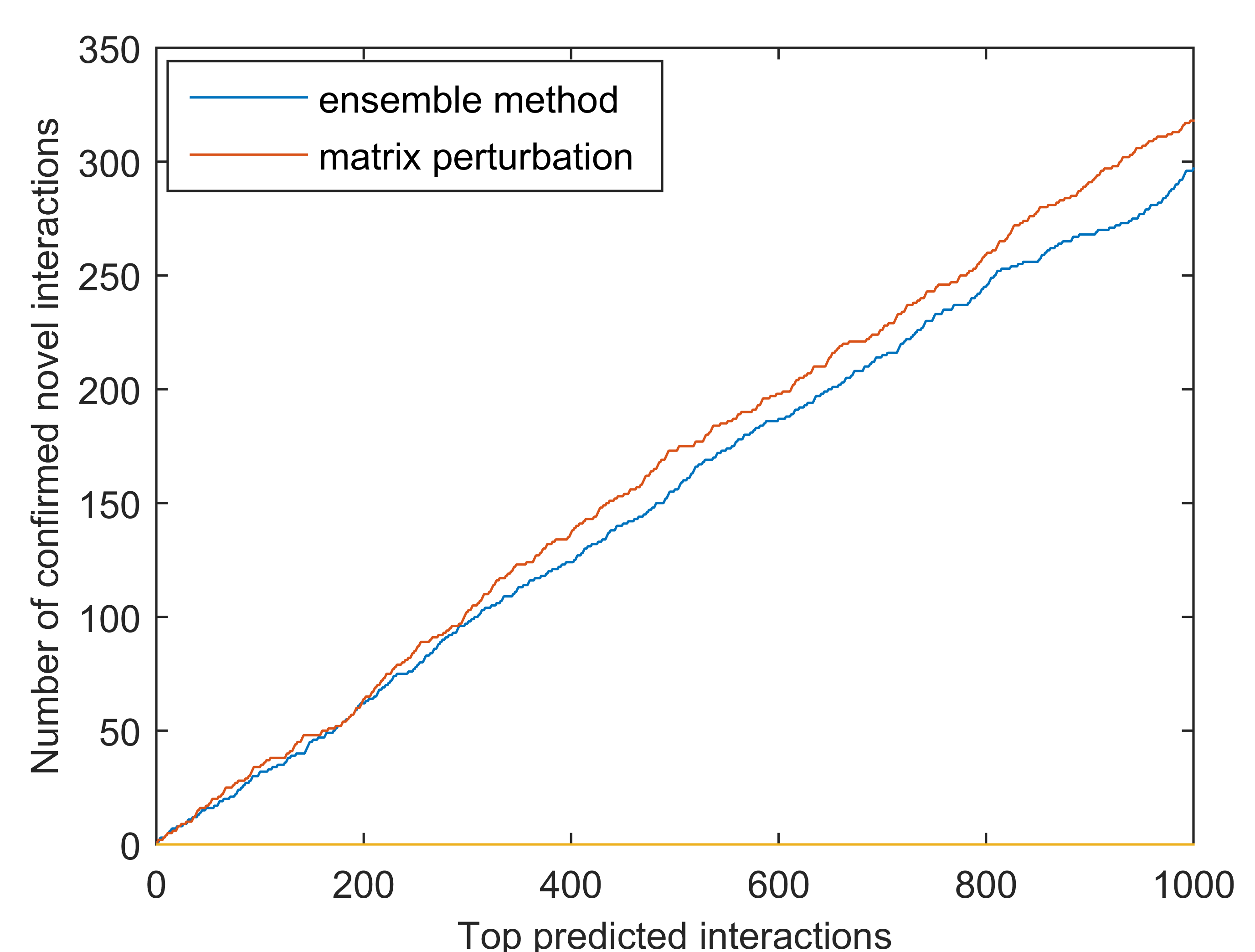

Supplement: Additional file 2: — Visualization of the number of predictions vs. number of confirmed interactions. (TIF 519 kb) [file 12859_2016_1415_MOESM2_ESM.tif]
